# Environmental and occupational respiratory diseases – 1058. Clinical and diagnostic characteristics of mycoplasma pneumoniae pneumonia in children with lobar pneumonia

**DOI:** 10.1186/1939-4551-6-S1-P56

**Published:** 2013-04-23

**Authors:** Jae Ho Lee, Eun Ae Yang, Jin Hwan Kim, Jae Ho Lee

**Affiliations:** 1 Department of Pediatrics, Chungnam National University School of Medicine, Daejeon, South Korea; 2 Department of Radiology, Chungnam National University Hospital, Daejeon, South Korea

## Background

*Mycoplasma pneumoniae* (*M. pneumoniae*) infected lobar pneumonia has increased recently in children in Korea.

## Objective

To evaluate the clinical and laboratory characteristics of lobar pneumonia infected by *M. pneumoniae* and to find more sensitive diagnostic tool in children.

## Methods

We analyzed medical records of 78 children, admitted to Chungnam National University Hospital and diagnosed with lobar pneumonia by chest X-rays between March 2010 and December 2011. White blood cells, C-reactive protein (CRP), procalcitonin (PCT), specific antibodies to *M. pneumoniae*, and cold agglutinin (CA) were measured at admission. Children were divided into 2 groups: those with *M. pneumoniae* infection (group A) and those without (group B). Group A children were also subdivided into 2 categories: those with increased CA (group A1) and those without (group A2).

## Results

*M. pneumoniae* infection usually occurred in summer and autumn. Group A children accounted for 75.6% (59/78) of all cases. The onset age was higher in group A than in group B (*P*=0.016). WBC counts and PCT values were higher in group B than in group A (*P*=0.015 and *P*=0.011). Radiologic findings showed that the lower lobe was most commonly involved without predilection for either side and that pleural effusion was present in 13.6% of all cases. The duration of fever before admission was longer in group A1 than in group A2 (*P*=0.019).

## Conclusions

The clinical symptoms and signs of lobar pneumonia caused by *M. pneumoniae* infection were more severe and can be accurately diagnosed using serum PCT values than using CRP values.

